# A Longitudinal Study of Families Formed Through Reproductive Donation: Parent-Adolescent Relationships and Adolescent Adjustment at Age 14

**DOI:** 10.1037/dev0000372

**Published:** 2017-07-31

**Authors:** Susan Golombok, Elena Ilioi, Lucy Blake, Gabriela Roman, Vasanti Jadva

**Affiliations:** 1 Centre for Family Research, University of Cambridge

**Keywords:** assisted reproduction, surrogacy, gamete donation, adolescence, parent-child relationships

## Abstract

The aim of the 6th phase of this longitudinal study was to establish whether children born through assisted reproduction involving reproductive donation were at risk for psychological problems following the transition to adolescence at age 14 and, if so, to examine the nature of these problems and the mechanisms involved. Eighty-seven families formed through reproductive donation, including 32 donor insemination families, 27 egg donation families, and 28 surrogacy families, were compared with 54 natural conception families. Standardized interviews, questionnaires, and observational assessments of the quality of parent-adolescent relationships and adolescent adjustment were administered to mothers, adolescents, and teachers. The mothers in surrogacy families showed less negative parenting and reported greater acceptance of their adolescent children and fewer problems in family relationships as a whole compared with gamete donation mothers. In addition, less positive relationships were found between mothers and adolescents in egg donation families than in donor insemination families as rated by both mothers and adolescents. There were no differences between family types for the adolescents themselves in terms of adjustment problems, psychological well-being, and self-esteem. Longitudinal analyses showed no differences between family types in negative parenting from age 7 to age 14, and a weaker association between negative parenting and adjustment difficulties for gamete donation than natural conception and surrogacy families. The findings suggest that the absence of a genetic link between mothers and their children is associated with less positive mother-adolescent relationships whereas the absence of a gestational link does not have an adverse effect.

Since the birth of the first baby through in vitro fertilization in 1978 (Steptoe & Edwards, 1978), more than 5 million children have been born through assisted reproductive technologies (Adamson, 2012), an increasing number of whom are born by reproductive donation, that is, by the donation of gametes (eggs or sperm) or the hosting of a pregnancy for another woman (surrogacy; Richards, Pennings, & Appleby, 2012). Children born through egg donation lack a genetic link with their mother whereas children born through sperm donation (donor insemination) lack a genetic link with their father. In the case of surrogacy, children lack a gestational link with their mother. Surrogacy children additionally lack a genetic link with their mother if the surrogate’s egg was used in their conception. The current study constitutes the sixth phase of the first longitudinal investigation of parenting and child development in families created through reproductive donation, and focuses on the children’s transition to adolescence when issues relating to identity and autonomy become salient and difficulties in parent-child relationships are most likely to arise (Smetana, Campione-Barr, & Metzger, 2006; Steinberg & Silk, 2002).

It has often been suggested that the creation of families through reproductive donation, whereby children lack a genetic and/or gestational relationship with their parents, may be detrimental to positive family functioning (Baran & Pannor, 1993; Daniels & Taylor, 1993; Velleman, 2005). This idea arose, in part, from studies of adoptive families, in which children similarly lack a biological connection to their parents. There is a large body of research demonstrating that adopted children show higher rates of emotional and behavioral problems than do nonadopted children (Palacios & Brodzinsky, 2010). However, recent meta-analyses have found these differences to be small, with the large majority of adopted children functioning within the normal range (Juffer & van IJzendoorn, 2005, 2007). In addition, the psychological problems shown by adopted children appear to be largely related to factors associated with the adoption, such as children’s experiences of abusive or neglectful parenting and multiple caretakers in the years before the adoption took place, rather than the absence of a biological link to their adoptive parents (Palacios & Brodzinsky, 2010).

However, the transition to adolescence presents specific challenges for adopted children. It has been shown that adopted adolescents need to integrate their experiences of being adopted into a meaningful narrative in order to develop a secure sense of identity (Grotevant & Von Korff, 2011). Moreover, adopted children show an increase in adjustment problems at adolescence (Fergusson, Lynskey, & Horwood., 1995; Sharma, McGue, & Benson, 1996; van der Voort et al., 2014; van der Voort, Linting, Juffer, Bakermans-Kranenburg, & van IJzendoorn, 2013). Although children conceived by gamete donation and surrogacy differ from adopted children in that they have a genetic link to one parent (their father in egg donation and surrogacy families and their mother in donor insemination families), and are raised by their mother and father from birth, the absence of a genetic and/or gestational connection to a parent is considered to create important similarities between children born through reproductive donation and adopted children which may have implications for their identity development, psychological adjustment, and relationships with their parents (Cahn, 2009).

Adoptive parents also face specific challenges when their children reach adolescence. Greater conflict has been found between adoptive parents and adopted adolescents than between nonadoptive parents and nonadopted adolescents (Rueter, Keyes, Iacono, & McGue, 2009). Moreover, poor communication about adoption has been associated with more negative relationships between adoptive parents and their adopted adolescents (Brodzinsky & Pinderhughes, 2002; Rueter & Koerner, 2008). Adoptive parents are therefore encouraged to acknowledge the difference between adoptive and biological families and create a family environment that supports open communication about adoption (Brodzinsky, 2011).

Research on stepfamilies, in which one parent is genetically unrelated to the child, has also given rise to the idea that the absence of a genetic link between parents and children may have an adverse effect on family relationships and children’s psychological adjustment. Stepfamilies, like adoptive families, are associated with raised levels of psychological problems for children (Dunn, Davies, O’Connor, & Sturgess, 2000; Dunn et al., 1998; Hetherington & Stanley-Hagan, 2002). This finding appears to be more marked in stepmother than in stepfather families (Dunn et al., 2000; Hetherington & Stanley-Hagan, 2002; O’Connor, Dunn, Jenkins, Pickering, & Rasbash, 2001). Once again, these difficulties appear to result from associated factors, such as disruption of the relationship with an existing parent and the acquisition of new family members, rather than the absence of a biological link between the stepparent and the child. Nevertheless, Dunn et al. (2000) reported that parents in families comprising both step and biological children were less affectionate toward, and less supportive of, their step than their biological children.

The earlier phases of the present longitudinal study were conducted when the children were at age 1 (Golombok, Lycett, et al., 2004a; Golombok, Murray, Jadva, MacCallum, & Lycett, 2004b), age 2 (Golombok, Jadva, Lycett, Murray, & MacCallum, 2005; Golombok, MacCallum, Murray, Lycett, & Jadva, 2006), age 3 (Golombok, Murray, Jadva, et al., 2006), age 7 (Golombok, Readings, Blake, Casey, Marks, et al., 2011; Golombok, Readings, Blake, Casey, Mellish, et al., 2011), and age 10 (Golombok, Blake, Casey, Roman, & Jadva, 2013). Contrary to the concerns that had been expressed regarding the potentially negative psychological consequences of reproductive donation, the differences identified between family types in the preschool years indicated more positive parent-child relationships in these families, irrespective of the type of reproductive donation used, than in the comparison group of natural conception families. The children themselves showed high levels of psychological adjustment but did not differ from the naturally conceived children in spite of their experience of highly involved parenting. In the middle school years, by which time children show an awareness of biological inheritance (Solomon, Johnson, Zaitchik, & Carey, 1996; Williams & Smith, 2010) and of the meaning and implications of the absence of a biological connection to parents (Brodzinsky, 2011), positive parent-child relationships prevailed, although the reproductive donation families no longer showed more positive parent-child relationships than did the natural conception families. With respect to the children, those born through surrogacy exhibited higher levels of adjustment difficulties than the naturally conceived children at age 7 but were below the cutoff for clinical problems and no longer differed from the natural conception children by age 10. This pattern is similar to that shown by transnationally adopted children (Stams, Juffer, Rispens, & Hoksbergen, 2000) and has been attributed to these children’s need to confront identity-related issues at a young age (Juffer & van IJzendoorn, 2005). Likewise, children born through surrogacy may be concerned with issues relating to identity at an early age (Golombok et al., 2013).

In spite of the generally positive outcomes shown by families formed through egg donation, donor insemination, and surrogacy up to the middle school years, the challenges of adolescence may be more pronounced in families created by reproductive donation than in natural conception families. Thus the aim of the present phase of the study was to establish whether families formed through assisted reproductive technologies involving reproductive donation are at risk for psychological problems following the children’s transition to adolescence and, if so, to examine the nature of these problems and the mechanisms involved. The study is founded upon a relational developmental systems approach as an underlying conceptual framework (Aldwin, 2014; Overton, 2015), whereby bidirectional relations between individuals, the family, and the wider social world are viewed as influential in development. More specifically, the study was guided by the theoretical and research literature on parenting showing that the quality of children’s relationships with their parents is associated with children’s psychological adjustment, such that positive aspects of parenting including warmth, sensitivity and acceptance are associated with positive child adjustment whereas conflict, hostility, and rejection are associated with more negative outcomes for children (Bornstein, 2002; Collins, Maccoby, Steinberg, Hetherington, & Bornstein, 2000; Lamb, 2012).

In line with this framework and the research literature on adoption and stepparenting, it was hypothesized that families formed through reproductive donation would show higher levels of difficulties in mother-adolescent relationships and adolescent adjustment problems than natural conception families, arising from the absence of a genetic and/or gestational connection between the adolescents and their parents. Differences were also hypothesized according to the specific type of reproductive donation used. Surrogacy families were predicted to show higher levels of problems in mother-adolescent relationships and adolescent adjustment than gamete donation families due to the absence of a gestational link with the mother, as adolescents conceived using donated gametes were born to the parents who raised them, whereas adolescents in families created by surrogacy were born to another woman who conceived them with the specific intention of relinquishing them to the intended parents. In addition, egg donation families were predicted to show higher levels of problems in mother-adolescent relationships and adolescent adjustment than donor insemination families due to the absence of a genetic link with the mother as opposed to the father. As mothers are more involved with their children on a day-to-day basis than are fathers (Lamb, 2010, 2012), the absence of a genetic link to the mother may be more detrimental to family functioning than the absence of a genetic link to the father. It may also be relevant, as mentioned above, that children are more likely to experience psychological difficulties when raised by stepmothers than by stepfathers.

Two further hypotheses were tested longitudinally using data from Phase 4 when the children were aged 7 years and Phase 5 when the children were aged 10 years. The first was that parenting difficulties would become more marked at adolescence in reproductive donation than in natural conception families as the absence of a biological connection is expected to produce greater challenges at adolescence than in middle childhood. For the reasons outlined above, it was also hypothesized that parenting difficulties would become more marked in surrogacy than in gamete donation families, and in egg donation than in donor insemination families. The second focused on the impact of parenting in middle childhood on adolescent adjustment. Based on a cross-sectional study of assisted reproduction families showing a weaker association between maternal hostility and child depression in genetically unrelated than in genetically related mother-child dyads (Harold et al., 2011), it was predicted that the long-term association between negative parenting and adolescent adjustment would differ according to biological relatedness such that there would be a weaker association between preadolescent parenting problems and adolescent adjustment problems in reproductive donation families than in natural conception families.

## Method

### Participants

At Phase 5 of the study, parents were asked for permission to contact them again for follow up (see Golombok, Lycett, et al., 2004 and Golombok, Murray, et al., 2004 for details of the initial recruitment of families to the study). Those who agreed were approached by telephone, letter, or email as close as possible to the child’s 14th birthday. The present phase of the study involved 87 families with a child born through reproductive donation including 32 families with a child born through donor insemination, 27 families with a child born through egg donation, and 28 families with a child born through surrogacy, and a comparison group of 54 families with a naturally conceived child, representing 91%, 84%, 90%, and 100%, respectively, of the number of donor insemination, egg donation, surrogacy, and naturally conceived families seen at Phase 5, and 89%, 84%, 86%, and 100%, respectively, of the number of donor insemination, egg donation, surrogacy, and naturally conceived families seen at Phase 4. These percentages include six families who participated at age 10 who had not taken part at age 7, and seven families who participated at age 14 who had not taken part at age 10. Of the 16 families who were lost to follow up between the present phase and the previous phase at age 10, six (37.5%) could not be traced, four (25%) actively withdrew, and the remaining six families (37.5%) were unable to participate due to other commitments but did not withdraw from the study. For ethical reasons, it was not possible to administer questionnaires or the observational assessment to adolescents who had not been informed of the method of their conception. Thus, 50 adolescents who were aware of the method of their conception (24 surrogacy adolescents, 16 egg donation adolescents, and 10 donor insemination adolescents) participated in the study, as well as 52 natural conception adolescents, representing 98% of eligible adolescents.

As shown in Table 1, there were no differences between family types in the age or gender of the children. A one-way analysis of variance (ANOVA) showed that the age of the mother differed significantly between family types, *F*(3, 137) = 12.42, *p* < .001, reflecting the older age of the egg donation (*M* = 53.66 years) and surrogacy (*M* = 51.60 years) mothers than the donor insemination (*M* = 48.93 years) and natural conception (*M* = 48.27 years) mothers. There was also a significant difference between family types for number of siblings in the family, χ^2^(6) = 20.40, *p* < .01, with a greater number of siblings in the natural conception than in the reproductive donation families. Of the surrogacy families, 10 (35.7%) mothers were genetically related to their children as they used their own eggs to create the pregnancy.[Table-anchor tbl1]

Excluding the two families where the father had died, 83.5% of parents were still married or cohabiting at the time of the study. There was no significant difference between family types in the proportion of mothers who had separated or divorced from the child’s father, although there was a nonsignificant trend toward higher relationship breakdown among the surrogacy and donor insemination families alongside a particularly low rate among the egg donation families, χ^2^(3) = 6.42, *p* = .09. There was a significant difference between family types in mothers’ educational level, χ^2^(3) = 15.16, *p* = .002. The natural conception mothers had the highest rate of university degrees and the lowest rate was found among the surrogacy mothers. There was no difference in mothers’ ethnic group between family types. Ninety-two percent of mothers were White, with the remaining 8% identifying as Black or Asian.

### Procedure

A psychologist trained in the study techniques visited the families at home. Written informed consent to participate in the investigation was obtained from the mother. Mothers and adolescents also gave written informed consent for the adolescents to participate. Ethical approval for the study was granted by the University of Cambridge Psychology, Research Ethics Committee. The mothers were administered a standardized interview that was digitally recorded. In addition, the mothers and adolescents completed standardized questionnaires and participated together in a video-recorded observational task that lasted 5–10 min. The adolescents’ teachers completed a questionnaire to give an independent assessment of the adolescents’ adjustment. As data were obtained by interview on issues relating to the child’s conception, it was not possible for interviewers to be blind to family type. However, a section of the interview on the child’s psychological adjustment was rated by a child psychiatrist who was unaware of the method of the child’s conception.

### Measures

#### Mother-child relationships

##### Interview with mother

The mothers were interviewed using an adaptation of a semistructured interview designed to assess the quality of the mother-child relationship that has been validated against observational ratings of mother-child relationships in the home (Quinton & Rutter, 1988) and has been used successfully in previous studies of assisted reproduction families (Golombok et al., 2013; Golombok, Readings, Blake, Casey, Marks, et al., 2011, Golombok, Readings, Blake, Casey, Mellish, et al., 2011). Detailed accounts are obtained of the child’s behavior and the mother’s response to it, with particular reference to interactions relating to warmth and control. A flexible style of questioning is used to elicit sufficient information for each variable to be rated by the researcher using a standardized coding scheme based upon a detailed coding manual. Thus ratings are carried out by the researcher using in-depth information obtained from the mother rather than by the mother herself.

The following variables were coded at Phases 4, 5, and 6 of the study: (a) *expressed warmth* from 1 (*little*) to 5 (*high expressed warmth*) took account of the mother’s tone of voice, facial expressions, and gestures in addition to what the mother said about the child; (b) *sensitive responding* from 1 (*low*) to 4 (*high*) represented the mother’s ability to recognize and respond appropriately to her child’s needs; (c) *quality of interaction* from 1 (*low*) to 4 (*very high*) was based on the extent to which the mother and child wanted to be with each other and enjoyed each other’s company; (d) *frequency of battles* from 0 (*never/rarely*) to 5 (*a few times daily*) assessed the frequency of mother-child conflict; (e) *level of battles* from 0 (*none*) to 3 (*major*) assessed the severity of mother-child conflict; and (f) *resolution* from 0 (*full resolution*) to 3 (*no resolution*) assessed the attempt made to resolve the conflict. To establish interrater reliability, 47 randomly selected interviews were coded by a second interviewer and the interclass correlation coefficients were as follows: expressed warmth, .70; sensitive responding, .56; quality of interaction, .79; frequency of battle, .99; level of battle,.96; and resolution, .88.

##### Index of Family Relationships (IFR)

Mothers and adolescents completed this 25-item questionnaire designed to measure problems in family relationships (Hudson, 1989). The total score, which ranges from 0 to 100, gives an assessment of family relationship difficulties, with higher scores representing greater difficulties. Internal consistencies for the original sample ranged from 0.91 to 0.98, and for the present sample are .91 and .94 for the mother and adolescent questionnaires, respectively. The IFR has been found to show good discriminant validity and to distinguish between families with and without clinical problems.

##### Parental Acceptance Rejection Questionnaire (PARQ)

The short 24-item version of this questionnaire was administered to both mothers and adolescents to provide total scores of maternal acceptance/rejection comprising subscales of warmth/affection, hostility aggression, indifference/neglect, and undifferentiated rejection (Rohner, 2001). Mothers completed the questionnaire regarding their feelings toward their adolescents and the adolescents completed the questionnaire regarding their perceptions of their mothers’ feelings toward them. Thus data on maternal acceptance/rejection was obtained from both mothers and adolescents. Higher scores represent greater rejection whereas lower scores represent greater acceptance, with scores above 60 representing higher levels of rejection than acceptance. The PARQ has been reported to have good internal consistency, with alpha values of 0.91 and 0.84, respectively, for the parent and adolescent versions. The alpha values for the current study were .66 and .82, respectively, for the parent and adolescent versions, with the discrepancy for the parent version resulting from high levels of maternal acceptance in the present study.

##### Parental Control Scale (PCS)

This 13-item measure of parental control was completed by mothers and adolescents to provide total scores of behavioral control (Rohner, 2001). Mothers completed the questionnaire regarding the control they enforced on their adolescents and the adolescents completed the questionnaire regarding their perceptions of the control their mothers enforced on them. Thus data on maternal control was obtained from the perspectives of both mothers and adolescents. Higher scores reflected higher levels of behavioral control, with scores ranging from 13–26 indicating low control, 27–39 indicating moderate control, 40–45 indicating firm control, and 46–52 indicating restrictive control. The PCS has been shown to have good internal consistency with an average alpha of 0.73 from a meta-analysis of studies using this measure (Rohner & Khaleque, 2003). The alphas for the current sample were .75 and .84 for mothers and adolescents, respectively.

##### Mother-adolescent interaction

Mothers and adolescents participated together in a video-recorded observational assessment involving a vacation planning task in which they were given 5 min to plan a 2-week family holiday for which they had unlimited funds (Grotevant & Cooper, 1985). Mother-adolescent dyads were instructed to talk freely about whom they wished to go on the holiday, where they wished to go, and what they planned to do while there. The session was coded using the Parent-Child Interaction System (Deater-Deckard & Petrill, 2004) to assess the construct of mutuality, that is, the extent to which the mother and child engaged in positive dyadic interaction characterized by warmth, mutual responsiveness, and cooperation. The following variables were rated on a 7-point scale ranging from 1 (*no instances*) to 7 (*constant, throughout interaction*): (a) *mother’s responsiveness to child* assessed the extent to which the mother responded immediately and contingently to the child’s comments, questions, or behaviors; (b) *child’s responsiveness to mother* assessed the extent to which the child responded immediately and contingently to the mother’s comments, questions, or behaviors; (c) *dyadic reciprocity* assessed the degree to which the dyad showed shared positive affect, eye contact, and a “turn-taking” (conversationlike) quality of interaction; and (d) *dyadic cooperation* assessed the degree of agreement about whether and how to proceed with the task. To establish interrater reliability, 47 randomly selected interviews were coded by two raters who were unaware of family type. The intraclass correlations for child’s responsiveness to mother, dyadic reciprocity, and dyadic cooperation were 0.61, 0.71, and 0.69, respectively. It was not possible to calculate an intraclass correlation for mother’s responsiveness to child due to the restriction of range of the scores as most dyads obtained scores at the top end of the scale.

#### Children’s psychological adjustment

##### Strengths and Difficulties Questionnaire (SDQ)

The presence of adolescent psychological problems was assessed with the SDQ (Goodman, 2001) administered to mothers and adolescents. The SDQ produces an overall score of adolescent adjustment with scores of 13 or below classified as within the normal range, scores of 14–16 classified as borderline and scores of 17 or above classified as abnormal, that is, indicating psychological disorder. An independent assessment of the adolescents’ psychological adjustment was obtained by administering the SDQ to teachers. Following permission from the mother, the questionnaire was mailed to the adolescent’s teacher with an enclosed stamped addressed envelope for return to the researcher. Teachers were informed by covering letter that their responses to the questionnaire would not be reported back to the adolescent’s family or school. For teachers’ questionnaires, scores of 11 or below are classified as within the normal range, scores of 12–15 are classified as borderline, and scores of 16 or above are classified as abnormal.

The SDQ has been shown to have good internal consistency, test-retest and interrater reliability, and concurrent and discriminative validity (Goodman, 2001). For example, based on an epidemiological sample of more than 10,000 children in the United Kingdom (Goodman, 2001), internal consistency (Cronbach’s alpha) was found to be 0.73, test–retest reliability after 4–6 months was 0.62, and, in terms of validity, scores above the 90th percentile predicted a substantially raised probability of independently diagnosed psychiatric disorders. Internal consistencies for mothers, adolescents, and teachers, respectively, in the current study were .69, .77, and .62. In a review of the reliability and validity of the SDQ based upon 48 studies involving more than 130,000 children, Stone, Otten, Engels, Vermulst, and Janssens (2010) found the psychometric properties of the SDQ to be strong.

##### Rosenberg Self-Esteem Scale

The Rosenberg Self-Esteem Scale was administered to adolescents to provide a measure of overall self-worth (Rosenberg, 1979). This 10-item questionnaire ranging from 10 to 30, for which higher scores represent higher self-esteem and scores below 15 suggest low self-esteem, has been shown to have high internal consistency, with an average alpha of 0.81 across studies in different nations (Schmitt & Allik, 2005). Cronbach’s alpha was .89 for the present sample. In addition, the scale has been found to be negatively correlated with anxiety and depression (Torrey, Mueser, McHugo, & Drake, 2000) thus demonstrating construct validity.

##### Engagement, Perseverance, Optimism, Connectedness, and Happiness Measure of Adolescent Wellbeing (EPOCH)

The 20-item EPOCH was administered to adolescents to produce a total score of positive psychological functioning ranging from 20 to 100, with higher scores representing more positive functioning (Kern, Benson, Steinberg, & Steinberg, 2016). Although this is a new measure and, as such, lacks a body of data on its psychometric properties, it was administered to assess positive functioning in adolescence as opposed to the absence of psychological problems. Test–retest reliability has been shown to be satisfactory across 3 weeks, ranging from .55 for Connectedness to .61 for Happiness, and internal consistency has been found to be high, ranging from .85 to .95 in different samples. For the present sample, Cronbach’s alpha was .89. EPOCH scores have been shown to be negatively correlated with measures of emotional distress and behavior problems indicating that the EPOCH is a valid measure of adolescent well-being.

##### Ratings of psychiatric disorder

The presence of adolescent psychiatric disorder was assessed during the interview with the mother using a standardized procedure (Rutter, Cox, Tupling, Berger, & Yule, 1975). Detailed descriptions were obtained of any emotional or behavioral problems shown by the adolescent. These descriptions of actual behavior, which included information about where the behavior was shown, severity of the behavior, frequency, precipitants, and course of the behavior over the past year, were transcribed and rated by a child psychiatrist who was unaware of the nature of the study. A high level of reliability (*r* = .85) between ratings made by social scientists and those made “blindly” by a child psychiatrist has been demonstrated for this procedure and validity has been established through a high level of agreement between interview ratings of children’s psychological problems and mothers’ assessments of whether or not their children had emotional or behavioral difficulties (Rutter et al., 1975). Psychological problems, when identified, were rated according to severity on a 3-point scale ranging from 0 (*no disorder*) through 1 (*slight disorder*) to 2 (*marked disorder*) and type (emotional disorder, conduct disorder, mixed emotional and conduct disorder, developmental disorder, ADHD, psychotic disorder, or other disorder).

### Analysis Plan

In the first instance, confirmatory factor analysis was conducted with the interview variables relating to parenting quality (comparative fit index [CFI] = 1.00; Tucker-Lewis index [TLI] = 1.00; root-mean-square error of approximation [RMSEA] = .03, 90% confidence interval [CI] = [.00, .11]). Two factors were obtained, each with item loadings of at least 0.43. The first factor (comprising expressed warmth, sensitive responding, and quality of interaction) was labeled positive parenting and the second factor (comprising frequency of battles, level of battles, and resolution) was labeled negative parenting. The correlation between the two factors was *r* = −.37, *p* = .001, showing a slight negative relation between them. Comparisons between the surrogacy, egg donation, donor insemination, and natural conception families at age 14 were conducted using univariate and multivariate analyses of variance. Where significant overall differences were found between family types, the following Helmert contrasts were carried out: reproductive donation families versus natural conception families (RD vs. NC) to establish whether there were differences between families where children lacked a genetic and/or gestational relationship with their parents and families with biologically related children, surrogacy families versus gamete (egg and sperm) donation families (S vs. GD) to establish whether families with children who lacked a gestational relationship with their mother differed from families where mothers had given birth to their children, and egg donation families versus donor insemination families (ED vs. DI) to establish whether families where children lacked a genetic link to their mother differed from families where children lacked a genetic link to their father. Effect sizes for the Helmert contrasts were calculated using Cohen’s *d*. For the comparisons between the reproductive donation and natural conception families, the sample size was large enough to detect an effect size of .34 for a power of 0.80, and for the comparisons between the surrogacy and gamete donation families, and between the egg donation and donor insemination families, it was possible to detect effect sizes of .45 and .52, respectively for a power of 0.80. As the demographic variables that differed significantly between groups (mothers’ age, number of siblings in the family, and mothers’ educational level) were not correlated with the dependent variables, these were not entered into the analyses as covariates.

For the longitudinal analysis of parenting difficulties over time, a mixed analysis of variance with Helmert contrasts was used to investigate differences between family types in negative parenting between age 7 and age 14. Path analysis was used to examine the relation between negative parenting and child adjustment over time. All families who had data at a minimum of one time point were included, producing an enhanced sample of 165 families. First, longitudinal confirmatory factor analysis with measurement invariance constraints was applied across the negative parenting variables obtained at child ages 7, 10, and 14 to ensure the creation of robust measures that functioned equivalently over time. The negative parenting factor achieved partial measurement equivalence and excellent fit to the data (CFI = .95; TLI = .96; RMSEA = .04, 90% CI [.00, .07]). The path analysis first focused on examining stability in negative parenting over time. The relation between negative parenting and child adjustment was then tested through the inclusion of mothers’ SDQ scores at age 14 as an outcome variable in the model. The models were conducted using Mplus v.7.4. Model fit was considered excellent for CFI and TLI values ≥.95 and acceptable for CFI and TLI values ≥.90. Although the RMSEA is reported for the models (adequate fit is achieved for RMSEA ≤.08 and excellent fit is achieved for RMSEA ≤.06), it was not used for the evaluation of model fit as the RMSEA underperforms in small samples.

## Results

### Comparisons Between the Surrogacy, Egg Donation, Donor Insemination, and Natural Conception Families at Age 14

#### Mother-child relationships

As shown in Table 2, the positive parenting factor scores and the negative parenting factor scores from the interview with mothers were entered into a multivariate analysis of variance (MANOVA) with family type as the between-subjects factor. Wilks’ λ was significant, *F*(6, 272) = 2.90, *p* < .01. One-way ANOVAs found a significant difference between groups for negative parenting, *F*(3, 137) = 4.10, *p* < .01, but not for positive parenting. With respect to the questionnaires, the mothers’ and adolescents’ IFR scores were entered into a MANOVA with family type as the between-subjects factor. Wilks’ λ was significant, *F*(6, 184) = 5.11, *p* < .001. One-way ANOVAs identified a significant difference between groups for both mothers’ scores, *F*(3, 93) = 10.16, *p* < .001, and adolescents’ scores, *F*(3, 93) = 2.92, *p* < .05. In addition, the mothers’ and adolescents’ total acceptance/rejection scores on the PARQ were entered into a MANOVA with family type as the between-subjects factor. Wilks’ λ was significant, *F*(6, 184) = 3.06, *p* < .05. One-way ANOVAs identified a significant difference between groups for mothers’ scores, *F*(3, 93) = 5.42, *p* < .01, but not for adolescents’ scores. The mothers’ and adolescents’ total scores on the PCS were also entered into a MANOVA with family type as the between-subjects factor. Wilks’ λ was not significant, *F*(6, 180) = 0.56, *p* = *ns*, showing that there was no difference in maternal control between family types as rated by mothers or adolescents. Finally, the variables relating to the construct of mutuality from the observational assessment of mother-adolescent interaction (mother responsiveness, child responsiveness, dyadic reciprocity, and dyadic cooperation) were entered into a MANOVA with family type as the between-subjects factor. Wilks’ λ was not significant, *F*(12, 211) = 1.49, *p* = *ns*, showing that there was no difference in mother-adolescent interaction between family types.[Table-anchor tbl2]

In order to test the first hypothesis that families in which children lacked a genetic and/or gestational relationship with their parents would show higher levels of difficulties in mother-adolescent relationships than families with biologically related children, contrasts between the reproductive donation families and the natural conception families were carried out for the variables that showed an overall difference between family types. None of these contrasts was significant showing that the reproductive donation families did not differ from the natural conception families with respect to the quality of mother-adolescent relationships.

In terms of the second hypothesis that families with children who lacked a gestational relationship with their mother would show higher levels of difficulties in mother-adolescent relationships than families in which mothers had given birth to their children, contrasts between surrogacy families and gamete donation families were carried out for the variables that differed between family types. The level of negative parenting was found to be significantly lower in the surrogacy families than in the gamete donation families (S vs. GD, *p* < .01, *d* = .77). In addition, the mothers’ IFR scores were lower in the surrogacy families compared with the gamete donation families (S vs. GD, *p* < .001, *d* = .77), reflecting lower levels of family relationship difficulties in the surrogacy families. There was no difference between the surrogacy families and the gamete donation families for the adolescents’ scores. Similarly, the contrasts for the PARQ showed lower scores in the surrogacy families compared with the gamete donation families for mothers (S vs. GD, *p* < .05, *d* = .61), reflecting greater parental acceptance in the surrogacy families, but not for adolescents.

Regarding the third hypothesis that families in which children lacked a genetic link to their mother would show higher levels of difficulties in mother-adolescent relationships than families in which children lacked a genetic link to their father, contrasts between egg donation families and donor insemination families were carried out for the variables that differed between family types. For the IFR, higher scores were found in the egg donation families compared with the donor insemination families for both mothers and adolescents (ED vs. DI, *p* < .01, *d* = .46 for mothers; ED vs. DI, *p* < .05, *d* = .89, for adolescents), reflecting greater family relationship difficulties in the egg donation families. Similarly, for the PARQ, mothers and adolescents from egg donation families obtained higher scores than mothers and adolescents from donor insemination families (ED vs. DI, *p* < .01, *d* = .59 for mothers; ED vs. DI, *p* < .05, *d* = .91 for adolescents), reflecting lower parental acceptance in the egg donation families.

#### Adolescent adjustment

One-way ANOVAs with family type as the between-subjects factor were carried out for the SDQ separately for the mothers’ and teachers’ scores in order to maximize the number of participants in each analysis. There were no significant differences between family types for either mothers or teachers (Table 3). Although only just over half of the teachers completed the SDQ, there was no significant difference in mothers’ total SDQ scores between those adolescents for whom teachers’ SDQ scores were available and those for whom they were not, and no significant difference between family types in the proportion of teachers who did not complete this questionnaire.[Table-anchor tbl3]

The adolescents’ scores on the EPOCH, the SDQ, and the Rosenberg Self-Esteem Scale were entered into a MANOVA with family type as the between-subjects factor. Wilks’ λ was not significant, *F*(9, 216) = 0.45, *p* = *ns*, showing that there was no difference in adolescents’ adjustment between family types as rated by the children themselves.

Thus, there was no evidence in support of the first hypothesis that adolescents who lacked a genetic and/or gestational relationship with their parents would show higher levels of adjustment difficulties than those who were biologically related to their parents. Neither was there evidence for the second hypothesis that adolescents who lacked a gestational relationship with their mother would show higher levels of adjustment difficulties than those whose mothers had given birth to them, or for the third hypothesis that adolescents who lacked a genetic connection to their mother would show higher levels of adjustment difficulties than those who lacked a genetic connection to their father.

The child psychiatrist identified psychiatric disorder among four (14.3%) surrogacy adolescents (2 with emotional disorder and 2 with developmental disorder), three (11.5%) egg donation adolescents (2 with emotional disorder and 1 with epilepsy), three (9.7%) donor insemination adolescents (2 with emotional disorder and 1 with mixed emotional and conduct disorder), and one (1.9%) naturally conceived adolescent (with emotional disorder). There was no difference between family types in the presence of psychiatric disorder, χ^2^(3) = 4.88, *p = ns.* However, there was a significant difference between family types when psychiatric disorder was subdivided into slight and marked disorder, χ^2^(6) = 14.54, *p* < .05, reflecting a higher proportion of adolescents in surrogacy families showing a marked disorder (10.7%) compared with 1.9% of adolescents in natural conception families and none in egg donation or donor insemination families.

### Longitudinal Analysis of Negative Parenting From Age 7 to Age 14

The variable negative parenting was entered into a mixed ANOVA with Helmert contrasts, with time as the within-subjects factor and family type as the between-subjects factor. Wilks’ λ was significant with respect to time, *F*(2, 160) = 993.47, *p* < .01, reflecting an increase in negative parenting between age 7 and age 10, followed by a decrease between age 10 and age 14. For the interaction between time and family type, Wilks’ λ was not significant, *F*(6, 320) = 0.89, *p* = *ns*, showing that change in negative parenting over time was similar for all family types.

### Longitudinal Analysis of the Association Between Negative Parenting and Adolescent Adjustment at Age 14

A path analysis whereby factor scores of negative parenting at each time point were used as predictors of factor scores of negative parenting at the subsequent time point revealed high stability across all time points in individual differences in negative parenting, with a similar pattern for all four family types. To investigate the influence of negative parenting on adolescent adjustment, the path analysis was modified to include mothers’ SDQ scores at age 14 which were regressed onto mothers’ negative parenting scores at age 14. In addition, to investigate long-term influences, indirect paths were specified from parenting at ages 10 and 7 to mothers’ SDQ scores at age 14, via parenting at age 14. As shown in Table 4, higher levels of adjustment difficulties at age 14 were explained by higher levels of negative parenting for naturally conceived adolescents and for adolescents conceived through surrogacy, but not for adolescents conceived by egg donation or donor insemination. Tests of indirect effects indicated the presence of adverse influences of negative parenting as early as 7 years beforehand, when the child was aged 7, as mothers who showed higher levels of negative parenting at the earlier time points maintained a higher level of negative parenting over time, relative to other mothers.[Table-anchor tbl4]

## Discussion

Despite the concern that children born through reproductive donation would be at risk for psychological difficulties at adolescence, the findings of the present phase of this longitudinal study of families formed through egg donation, donor insemination, and surrogacy showed that these families did not differ from natural conception families when the children reached age 14. The interview and observational ratings, as well as the scores on questionnaires for which norms were available, were characteristic of positive mother-adolescent relationships and well-adjusted adolescents. A possible explanation for this finding lies in the mothers’ high motivation to have children. Children born through reproductive donation are, by necessity, planned and there is evidence to show that planned pregnancies are associated with more positive psychological outcomes for mothers and children (Carson et al., 2013; Nelson & O’Brien, 2012), with a longitudinal study of adolescents born as a consequence of unplanned pregnancies showing raised levels of externalizing problems at age 14 (Hayatbakhsh et al., 2011).

Where differences in the quality of mother-adolescent relationships were identified between family types, these reflected more positive relationships in the surrogacy families compared with the gamete donation families. The mothers in surrogacy families showed less negative parenting and reported greater acceptance of their adolescent children and fewer problems in family relationships as a whole. These findings are unexpected, given that surrogacy is considered to be the most controversial form of reproductive donation and has been assumed to carry the greatest psychological risks; not only are the children relinquished by the woman who gave birth to them but also negative societal attitudes toward surrogacy prevail (Anderson, 2000; Brazier, Campbell, & Golombok, 1998). However, surrogacy is a complex process that requires a trusting relationship between the intended parents and the surrogate, and the majority of couples who become parents in this way maintain contact with the surrogate as the child grows up (Jadva, Blake, Casey, & Golombok, 2012). Thus, surrogacy is not undertaken lightly and couples who have followed this route to parenthood are not only highly committed to becoming parents but also are willing to accept a third party into the process of forming a family. As surrogacy is not something that most prospective parents would contemplate even when faced with infertility, it is perhaps not surprising that their strong desire for a child translates into more positive parenting than that shown by parents of children conceived by gamete donation. It may also be relevant that 10 (35.7%) of the surrogacy mothers had used their own eggs to create the pregnancy and thus were genetically related to their children.

Differences were also identified between the egg donation families and the donor insemination families. These differences indicated less positive relationships between mothers and adolescents in egg donation families than in donor insemination families both in terms of mothers’ acceptance of their adolescents and the functioning of the family as a whole. Importantly, these differences were identified not only from the mothers’ reports but also from the adolescents’ reports which gives greater weight to the findings. Although it is essential to stress that the scores for both mothers and children in egg donation families are indicative of high levels of maternal acceptance and family functioning, inspection of the mean scores shows the donor insemination families to be similar to the natural conception comparison group whereas the egg donation families show less positive scores. This finding is in line with the hypothesis that the absence of a genetic link between mothers and their children would present more difficulties for mother-child relationships than would the absence of a genetic link between children and their fathers. Although there was no significant difference in the rates of divorce or separation between family types, it is notable that only 7.4% of egg donation families had separated or divorced. This is very low compared with the national divorce rate reported by the Office of National Statistics which is 28% for couples with children of this age and suggests that mothers who do not have a genetic tie to their children may be more likely to remain with their children’s father.

Regarding the adolescents, there were no differences between family types in emotional or behavioral problems as assessed by the SDQ completed by mothers, teachers, and the adolescents themselves. Neither were there differences in adolescent well-being or self-esteem. For all of these measures, the adolescents obtained scores that reflected high levels of psychological adjustment. The ratings of interview transcripts by a child psychiatrist who was unaware of the child’s family type were in line with these findings. The higher proportion of adolescents in surrogacy families who showed a marked disorder reflected developmental disorder in two of the three adolescents with this classification which is unlikely to be related to the quality of family relationships.

No differences were found for the observational measure of mother-adolescent interaction. Although this suggests that the groups did not differ in the quality of dynamic interactions between mothers and their adolescents, it is possible that the task of planning a family holiday was not sufficiently emotive or salient to elucidate differences in interaction patterns. The use of a conflict task was considered but was felt to be inappropriate with these particular families due to the sensitivities associated with the circumstances of the children’s birth. In terms of mothers’ level of control of their children, no difference was identified between family types.

Although it has been suggested that the additional challenges of adolescence may result in greater difficulties in parenting for families created by reproductive donation than for natural conception families, no differences were found between family types in negative parenting from middle childhood to adolescence. In all family types, negative parenting increased between age 7 and age 10 and then decreased between age 10 and age 14. This finding is consistent with a meta-analysis of parent-child conflict over time which concluded that conflict peaks during early adolescence (age 10–12) and then declines (Laursen, Coy, & Collins, 1998). In line with the hypothesis that there would be a weaker association between negative parenting and adjustment difficulties for genetically unrelated than genetically related mother-adolescent dyads, an association was found between negative parenting and adjustment difficulties at age 14 for naturally conceived adolescents but not for adolescents conceived by egg donation or donor insemination. This finding is consistent with the view that the association between parenting and child adjustment is, to some extent, genetically transmitted (Harold et al., 2011; Rutter, 2006). Although the surrogacy families were similar to the natural conception families in showing an association between negative parenting and adolescent adjustment difficulties at age 14, this finding may result from the presence of a genetic connection between one third of the surrogacy mothers and their children. It should be noted that differences between family types with respect to the presence of a relation between maternal negativity and child behavior problems were inferred based on the pattern of significant and nonsignificant findings.

A limitation of the study is the small sample size. As a consequence, differences between family types may not have been detected. Nevertheless, consistent differences were identified from different informants even with this small sample size, with large and medium effect sizes for the IFR and the PARQ. A further limitation was the low response rate (51%) from teachers which may have biased the findings to the extent that teachers may have been reluctant to complete questionnaires for adolescents who were showing emotional or behavioral problems. However, 40 (28.3%) of the teachers were not approached because the mothers did not give permission and thus the response rate from teachers who were asked to complete the questionnaire was 71.2%. In addition, there was no difference in the proportion of missing teachers’ questionnaires between family types and no difference in mothers’ SDQ scores between families with and without teachers’ questionnaires. Sampling bias may also have arisen from families lost to follow up since the previous phase of the study. However, the retention rate was high and attrition did not differ according to family type. A further limitation of the small sample was that the surrogacy families could not be divided into subsamples of families with genetically related, and genetically unrelated, children.

The study had a number of advantages. In particular, this is the only longitudinal study worldwide of parenting and child development in surrogacy families and, with the exception of a previous study by the same research team, the only longitudinal study of parenting and child development in egg donation and donor insemination families. Moreover, it is the first study to obtain data from the children themselves. In addition, the ratings of adolescent psychiatric disorder by a child psychiatrist who was “blind” to family type, as well as the teachers’ questionnaires, provided independent validation of the data obtained from mothers and adolescents. A further advantage is that parenting quality was assessed using the same interview procedure at three time points from age 7 to age 14 which enabled longitudinal analyses of negative parenting and its association with adolescent adjustment to be carried out.

From a theoretical perspective, studying families where children lack a biological connection to their parents can increase understanding of the importance of biological relatedness for parent-child relationships and child adjustment. More specifically, studying families formed through reproductive donation can help establish whether the lack of a genetic and/or gestational link between parents and their children has an adverse impact on parenting or adolescent adjustment in the absence of the potentially confounding factors associated with adoption and living in a stepfamily. Overall, the findings of this longitudinal study of children born through egg donation, donor insemination, and surrogacy did not indicate raised levels of mother-adolescent relationship difficulties or adolescent adjustment problems compared with natural conception families. However, the differences identified between the egg donation and donor insemination families suggest that the absence of a genetic link between mothers and their children is associated with less positive mother-adolescent relationships than is the absence of a genetic link between the father and the child. In contrast, it appears that the absence of a gestational link between mothers and their children does not have an adverse effect on the quality of mother-child relationships at adolescence.

## Figures and Tables

**Table 1 tbl1:** Sociodemographic Information by Family Type

	Natural conception		Surrogacy		Egg donation		Donor insemination		*F*	*p*
Variable	Mean	*SD*	Mean	*SD*	Mean	*SD*	Mean	*SD*		
Age of mother (years)	48.27	2.73	51.60	4.85	53.66	5.91	48.93	3.45	12.42	.000
Age of children (months)	169.16	4.23	167.17	5.84	168.29	5.73	167.37	5.04	1.32	*ns*
	*N* (%)		*N* (%)		*N* (%)		*N* (%)		χ^2^	*p*
Child’s gender									1.69	*ns*
Boy	25 (46.3)		12 (42.9)		15 (55.6)		18 (56.3)			
Girl	29 (53.7)		16 (57.1)		12 (44.4)		14 (43.8)			
Number of siblings									20.40	.002
0	4 (7.4)		12 (42.9)		13 (48.1)		11 (34.4)			
1	41 (75.9)		13 (46.4)		11 (40.7)		17 (53.1)			
2+	9 (16.7)		3 (10.7)		3 (11.1)		4 (12.5)			
Marital Status									6.42	.09
Married	48 (88.9)		21 (75)		25 (92.6)		22 (73.3)			
Separated/divorced	6 (11.1)		7 (25.0)		2 (7.4)		8 (26.7)			
Ethnic group:									2.90	*ns*
White	47 (97.9)		25 (100)		23 (92.0)		26 (96.3)			
Non-white	1 (2.1)		0 (0)		2 (8)		1 (3.7)			
Mothers’ educational level									15.16	.002
No university degree	19 (35.2)		22 (78.6)		16 (59.3)		19 (59.4)			
University degree	35 (64.8)		6 (21.4)		11 (40.7)		13 (40.6)			

**Table 2 tbl2:** Means, SDs, F, and p Values for Comparisons of the Interview, Questionnaire, and Observational Assessment of the Quality of Parent-Adolescent Relationships Between Family Types

											Contrasts					
	Natural conception		Surrogacy		Egg donation		Donor insemination		*F*	*p*	NC vs RD		S vs GD		ED vs DI	
Variable	Mean	*SD*	Mean	*SD*	Mean	*SD*	Mean	*SD*			*p*	*d*	*p*	*d*	*p*	*d*
Quality of parenting									2.90	.009						
Positive parenting^a^	−.10	.87	.03	.86	.04	.66	−.13	.71	.43	*ns*	*ns*	.09	*ns*	.11	*ns*	.26
Negative parenting^b^	−.09	.70	−.19	.58	.26	.53	.15	.40	4.10	.008	*ns*	.49	.003	.77	*ns*	.23
Index of family relationships^b^									5.11	.000						
Mother	11.61	7.93	6.37	4.99	20.86	12.11	10.80	3.91	10.16	.000	*ns*	.09	.000	.77	.002	.46
Adolescent	14.32	9.93	12.96	7.56	21.30	14.87	10.61	8.09	2.92	.038	*ns*	.05	*ns*	.36	.012	.89
Parental acceptance/Rejection questionnaire^b^									3.06	.007						
Mother	29.32	4.26	27.28	2.70	32.50	5.92	27.40	2.54	5.42	.002	*ns*	.01	.034	.61	.003	.59
Adolescent	28.78	4.95	27.61	3.42	30.18	5.69	26.10	2.72	1.92	*ns*	*ns*	.23	*ns*	.22	.031	.91
Parental control scale^b^									.56	*ns*						
Mother	35.14	4.86	35.95	3.70	36.93	5.93	34.80	3.29	.69	*ns*	*ns*	.24	*ns*	.05	*ns*	.16
Adolescent	33.42	6.94	35.50	7.23	33.66	6.59	33.10	6.85	.48	*ns*	*ns*	.14	*ns*	.30	*ns*	.08
Observational assessment^a^									1.49	*ns*						
Mother responsiveness	6.07	1.02	6.09	.92	6.57	.51	6.29	.95	1.13	*ns*	*ns*	.22	*ns*	.48	*ns*	.36
Child responsiveness	6.02	.95	5.73	1.63	6.57	.64	6.43	1.13	1.81	*ns*	*ns*	.08	*ns*	.61	*ns*	.15
Dyadic reciprocity	4.57	1.64	4.68	1.58	3.86	.94	5.43	.97	1.85	*ns*	*ns*	.04	*ns*	.21	*ns*	.15
Dyadic cooperation	5.52	1.28	5.36	1.52	5.64	.84	6.29	1.11	.94	*ns*	*ns*	.07	*ns*	.39	*ns*	.66
*Note.* NC = natural conception; RD = reproductive donation (surrogacy, egg donation and donor insemination combined); S = surrogacy; GD = gamete donation (egg donation and donor insemination combined); ED = egg donation; DI = donor insemination.																
^a^ Higher scores represent fewer difficulties. ^b^ Higher scores represent greater difficulties.																

**Table 3 tbl3:** Means, SDs, F, and p Values for Comparisons of the Strengths and Difficulties Questionnaire Completed by Mothers and Teachers and the EPOCH, SDQ, and Rosenberg Self-Esteem Scale Completed by Adolescents Between Family Types

											Contrasts					
	Natural conception		Surrogacy		Egg donation		Donor insemination		*F*	*p*	NC vs RD		S vs GD		ED vs DI	
Variable	Mean	*SD*	Mean	*SD*	Mean	*SD*	Mean	*SD*			*p*	*d*	*p*	*d*	*p*	*d*
Strengths and Difficulties Questionnaire (SDQ)^b^																
Mothers’ ratings	4.47	3.71	5.61	3.85	5.70	2.75	4.76	3.19	1.04	ns	ns	.31	ns	.24	ns	.31
Teachers’ ratings	3.76	3.94	7.00	5.73	5.61	5.17	4.58	3.87	1.76	ns	ns	.22	ns	.44	ns	.22
Adolescent adjustment																
EPOCH^a^	3.72	.63	3.78	.54	3.64	.58	3.75	.29	.18	ns	ns	.23	ns	.02	ns	.23
SDQ^b^	9.55	4.74	9.00	5.23	10.75	5.90	8.40	3.94	.566	ns	ns	.47	ns	.02	ns	.47
Rosenberg Self-Esteem Scale^a^	22.81	5.17	24.05	4.75	22.75	6.93	22.40	6.20	.30	ns	ns	.05	ns	.01	ns	.05
*Note.* SDQ = Strengths and Difficulties Questionnaire; EPOCH = Engagement, Perseverance, Optimism, Connectedness and Happiness Measure of Adolescent Wellbeing; NC = natural conception; RD = reproductive donation (surrogacy, egg donation and donor insemination combined); S = surrogacy; GD = gamete donation (egg donation and donor insemination combined); ED = egg donation; DI = donor insemination.																
^a^ Higher scores represent fewer difficulties. ^b^ Higher scores represent greater difficulties.																

**Table 4 tbl4:** Path Analysis of the Influence of Negative Parenting on Mothers’ Strengths and Difficulty Questionnaire Scores

Variable	Natural conception	Surrogacy	Donor insemination	Egg donation
Stability in negative parenting over time				
Age 7 → Age 10	.87*** [.79, .95]	.90*** [.82, .97]	.86*** [.77, .95]	.88*** [.76, .97]
Age 10 → Age 14	.86** [.78, .93]	.89*** [.83, .94]	.84*** [.75, .94]	.90*** [.84, .97]
Prediction of SDQ scores at age 14 from negative parenting				
Age 14	.34** [.10, .59]	.29* [.01, .57]	.02 (*ns*) [−.28, .31]	.06 (*ns*) [−.34, .46]
Age 10 → Age 14	.29** [.01, .51]	.26* [.003, .51]	.01 (*ns*) [−.24, .26]	.05 (*ns*) [−.31, .42]
Age 7 → Age 10 → Age 14	.25** [.06, .45]	.23^†^ [−.001, .46]	.01 (*ns*) [−.20, .23]	.05 (*ns*) [−.27, .37]
	CFI	TLI	RMSEA	RMSEA 90% CI
Model fit	.98	.96	.14	[.00, .23]
*Note.* SDQ = Strengths and Difficulties Questionnaire; CFI = comparative fit index; TLI = Tucker-Lewis index; RMSEA = root-mean-square error of approximation; CI = confidence interval. The numbers in square brackets represent 95% confidence intervals.				
^†^ *p* = .051. * *p* ≤ .05. ** *p* ≤ .01. *** *p* ≤ .001.				
